# SHORTEN PREOPERATIVE FASTING AND INTRODUCING EARLY EATING ASSISTANCE IN RECOVERY AFTER GASTROJEJUNAL BYPASS?

**DOI:** 10.1590/0102-672020210003e1606

**Published:** 2022-01-05

**Authors:** Eduardo WENDLER, Paulo Afonso Nunes NASSIF, Osvaldo MALAFAIA, Jose Luzardo BRITES, José Guilherme Agner RIBEIRO, Laura Brandão DE PROENÇA, Maria Eduarda MATTOS, Bruno Luiz ARIEDE

**Affiliations:** 1 Postgraduate Program in Principles of Surgery, Mackenzie Evangelical Faculty of Paraná/Medical Research Institute, Curitiba, PR, Brazil; 2 Rocio Hospital, Campo Largo, PR, Brazil

**Keywords:** Fasting, Clinical evolution, Gastric bypass, Complication, Jejum, Evolução clínica, Derivação gástrica, Complicação

## Abstract

**Rational:**

The metabolic response to surgical trauma is enhanced by prolonged preoperative fasting, contributing to increased insulin resistance. This manifestation is more intense on the 1^st^ and 2^nd^ postoperative days and is directly proportional to the size of the operation.

**Aim:**

To compare whether preoperative fasting abbreviation and early postoperative refeeding associated with intraoperative and postoperative fluid restriction interfere in the evolution of patients undergoing gastrojejunal bypass.

**Methods:**

Eighty patients indicated for Roux-en-Y gastrojejunal bypass were selected. They were randomly divided into two groups: Ringer Lactate (RL) group, who underwent a 6 hours solids fasting, with the administration of 50 g of maltodextrin in 100 ml of mineral water 2 hours before the beginning of anesthesia; and Physiologic Solution (PS) group, who underwent a 12 hours solids and liquids fasting. Anesthesia was standardized for both groups. During the surgical procedure, 1500 ml of ringer lactate solution was administered in the RL and 2500 ml of physiological solution (0.9% sodium chloride) in the PS. In both groups, the occurrence of bronchoaspiration was analyzed during intubation, and the residual gastric volume was measured after opening the abdominal cavity. In the postoperative period in Group RL, patients started a liquid diet 24 hours after the end of the operative procedure; whilst for PS group, fasting was maintained for the first 24 hours, it was prescripted 2000 ml of physiological solution and a restricted liquid diet after 36 hours. Each patient underwent CPK, insulin, sodium, potassium, urea, creatinine, PaCO2, pH and bicarbonate dosage in the immediate postoperative period, and 48 hours later, the exams were repeated.

**Results:**

There were no episodes of bronchoaspiration and gastrojejunal fistulas in either group. In the analysis of the residual gastric volume of the PS and RL groups, the mean volumes were respectively 16.5 and 8.8, which shows statistical significance between the groups. In laboratory tests, there was no difference between groups in sodium; PS group showed a higher level of serum potassium (p=0.029); whilst RL group showed a higher urea and creatinine values; CPK values were even for both; PS group demonstrated a higher insulin level; pH was higher in PS group; sodium bicarbonate showed a significant difference at all times; PaCO2 values in RL group was higher than in PS. In the analysis of the incidence of nausea and flatus, no statistical significance was observed between the groups.

**Conclusions:**

The abbreviation of preoperative fasting and early postoperative refeeding of Roux-en-Y gastrojejunal bypass with the application of ERAS or ACERTO Project accelerated the patient’s recovery, reducing residual gastric volume and insulin level, and do not predispose to complications.

## RESUMO

**Racional:**

A resposta metabólica ao trauma cirúrgico é potencializada pelo jejum pré-operatório prolongado que contribui para o aumento da resistência à insulina. Esta manifestação é mais intensa no 1º e 2º dias de pós-operatório e é diretamente proporcional ao porte da operação.

**Objetivo:**

Comparar se a abreviação do jejum pré-operatório e a realimentação precoce no pós-operatório associado à restrição hídrica no trans e pós-operatório interferem na evolução dos pacientes submetidos ao bypass gastrojejunal.

**Métodos:**

Foram recrutados 80 pacientes indicados ao bypass gastrojejunal em Y-de-Roux. Eles foram distribuídos randomicamente em dois grupos: ringer lactato (RL) que fizeram jejum de 6 h para sólidos, administrando 50 g de maltodextrina em 100 ml de água mineral 2 h antes do início da anestesia e de soro fisiológico (SF) que fizeram jejum de 12 h para sólidos e líquidos. A anestesia foi padronizada para os dois grupos. Durante o procedimento operatório no RL foi administrado 1500 ml solução de ringer lactato e no SF 2500 ml de soro fisiológico (0,9% de cloreto de sódio). Em ambos os grupos foram analisados durante a intubação a ocorrência ou não de bronco-aspiração e mensurado o volume gástrico residual após abertura da cavidade abdominal. No pós-operatório do Grupo RL, os pacientes iniciaram dieta liquida após 24 h do término do procedimento operatório; no Grupo SF foi mantido jejum nas primeiras 24 h, prescrição de 2000 ml de soro fisiológico e início da dieta líquida restrita com 36 h. Cada paciente realizou no pós-operatório imediato, ainda na sala de cirurgia, a dosagem de CPK, insulina, sódio, potássio, ureia, creatinina, PaCO2, pH e bicarbonato e em 48 h repetiu-se a coleta destes exames.

**Resultados:**

Não houve episódios de broncoaspiração e fístulas gastrojejunais em ambos os grupos. Na análise do volume residual gástrico dos grupos SF e RL, as médias de volume foram respectivamente 16,5 e 8,8 apresentando significância estatística entre os grupos. Nos exames laboratoriais não houve diferença entre os grupos no sódio; nível sérico de potássio no SF foi maior (p=0,029); ureia e creatinina maiores no RL; CPK não apresentou diferenças; insulina no grupo SF foram maiores; pH foi maior no SF; bicarbonato de sódio evidenciou diferença significativa em todos o momentos; PaCO2 no RL foi maior. Na análise de incidência de náusea e flatos não foram observados significância estatística entre os grupos.

**Conclusões:**

A abreviação do jejum pré-operatório e a realimentação precoce no pós-operatório de bypass gastrojejunal em Y-de-Roux com a aplicação de programas como ERAS ou Projeto Acerto aceleram a recuperação do paciente, diminuindo o volume gástrico residual e o nível de insulina, e não predispõem complicações.

## INTRODUCTION

Obesity is a hard-treatment chronic disease that has become a global epidemic that causes health issues for individuals and society. Although there is evidence of metabolically healthy obesity^13^, there is consensus about its harms, especially about the cardiovascular risks present in obese individuals with fat deposition in the visceral region, as shown in some longitudinal studies^13^.

According to the WHO - World Health Organization - Obesity results from an individual’s positive energy balance, which leads to excessive accumulation of body fats that causes health problems, lower life quality, and decreased life expectancy^16^. Moreover, obesity is also classified based on the body mass index - BMI, calculated by the ratio of body weight and the height to the power of 2 (BMI=kg/m²).

Currently, the number of surgical procedures - resection and anastomosis of stomach and small intestine - for morbid obesity has increased significantly^8^ ^,^ ^14^. 

A six to eight hours fasting before these procedures was implemented when rudimentary anesthetic techniques were still used. This technique was recommended to prevent pulmonary complications associated with gastric contents aspiration during anesthesia (Mendelson’s syndrome). Another work from the 1950s extended the maximum limit of gastric content to 25 ml to ensure that there would be no risk of bronchoaspiration under anesthesia^2^ ^,^ ^3^. 

Another problem related to prolonged preoperative fasting is increased insulin resistance, which enhances the metabolic response to surgical trauma. This result is commonly observed on the first and the second day after surgery, and it is directly proportional to the operation complexity^13^. 

There is no consensus on the best perioperative care in bariatric surgery. Evidence-based protocols and guidelines are mechanisms that must be implemented in the medical routine, as they reduce the morbidity and mortality of the population^28^. Standardizing clinical practice to make it safer is a challenge since physicians often neglect it. Despite this, the Society of Recovery After Surgery has reached a broad evidence-based consensus, known as Enhanced Recovery After Surgery (ERAS).

Initially, the principles of the ERAS protocol were applied in colorectal operations, which contributed to a large number of studies in this area. Other surgical specialties have been adopting these concepts, and there is constant updating as researches are developed^9^.

Several multimodal protocol guidelines, based on randomized studies and meta-analyses, showed that, even in large operations, it is possible to abbreviate preoperative fasting for two hours by ministering carbohydrate-containing liquids. Moreover, early feeding on postoperative and reduced hospitalization time is also classified as safe by these studies^3^.

In 2005, the ACERTO Project (Acceleration of Postoperative Total Recovery), based on an extensive literature review on perioperative care, initiated a pioneering multimodal program in the national territory, which, from its inception, highlighted the importance of nutritional issues in the recovery of the surgical patient. Evidence-based medicine has shown that programs for accelerating postoperative recovery, along the lines of ACERTO, are safe, reduce postoperative complications and hospitalization time without increasing hospital readmission rates^3^.

The use of the ACERTO Project or ERAS protocol has shown that they are safe in bariatric surgery and can improve intraoperative and postoperative clinical conditions by reducing hospitalization time^14^.

This study aimed to define whether preoperative fasting abbreviation, early feeding, trans and postoperative fluid restriction interfere in the gastrojejunal bypass patient recovery.

## METHODS

This work consists of a randomized clinical assay in which 80 patients indicated for Roux-en-Y gastrojejunal bypass (BGYR) were recruited after approval by the Research Ethics Committee of Faculdade Evangélica Mackenzie do Paraná, accordingly to Resolution 466/12 CNS under report number 1,999,670. After the selection criteria were applied, the patients were randomized and divided into two groups. The operations were performed at a single institution - Hospital do Rocio, Campo Largo, PR, Brazil.

### Selection criteria

Those eligible for BGYR and who agreed to participate in the study have signed an informed consent term. Inclusion criteria to cooperate this research were: BMI >35 kg/m^2^ associated with hypertension and/or diabetes or BMI between 40 kg/m^2^ and 46 kg/m^2^, surgical time less than 120 min and procedure performed by the same surgical team.

This study did not include those with renal and hepatic insufficiency or dysfunction, coagulation disorders, heart disease, allergic to dipyrone or non-steroidal anti-inflammatory drugs and who require ICU monitoring.

### Groups distribution

Patients were randomly divided into two groups: ringer lactate (RL) and Physiological Solution (PS) groups. Randomization was performed using the Random® program, in which the number provided would correspond to the group in which each patient would fit.

### Surgical Procedures

#### Preoperative

Patients from the RL group underwent 6 hours of solid fasting, administering 50 g of maltodextrin in 100 ml of mineral water 2 hours before the beginning of anesthesia, whilst the PS group underwent 12 hours of solids and liquids fasting.

#### Transoperative

Anesthesia was equally regulated for both groups with an anesthetic induction of propofol (1.5 mg/kg), fentanyl (3 mcg/kg) and cisatracurium (0.15 mg/kg) and monitored in a conventional way. Analgesia was multimodal with dipyrone (2 g), ketorolac (30 mg) and epidural anesthesia with ropivacaine associated with morphine. Nausea and vomiting prevention was performed with ondansetron (8 mg), dexamethasone (4 mg) and alizapride (50 mg). The same surgical technique of RYGB was used in all patients and performed by the same surgical team.

During the operative procedure, 1500 ml of ringer lactate solution was administered in a closed system in the RL group, whilst 2500 ml of saline solution was in the PS group. In both groups, the occurrence of bronchoaspiration during intubation was analyzed, and the residual gastric volume after reaching the abdominal cavity was measured. For this last procedure, the anesthetist collected gastric fluid through the Fouchet tube and the surgeon assisted by manipulating the stomach, making it more effective and precise. In the surgery room and after the operation, each patient underwent exams of CPK, insulin, sodium, potassium, urea, creatinine, PaCO2, pH and bicarbonate. 

#### Postoperative

#### RL Group

In the 24 hours RL group postoperative period, patients started a liquid diet and infusion of 1000 ml of ringer lactate solution, 1000 ml of glucose solution, antibiotic prophylaxis (Kefazol 1 g 8/8h), analgesics and antiemetics, when needed. Laboratory tests were redone, and the presence of nausea and flatus were monitored during this time. In the 48 hours postoperative period, patients started a restricted liquid diet 36 hours after surgery and were prescripted 500 ml of ringer lactate, 500 ml of glucose solution, analgesics and antiemetics, if necessary. Once again, laboratory tests were redone, and nausea and flatus were monitored during this time.

#### PS Group

In the 24 hours PS group postoperative period, patients maintained fasting and were prescripted 2000 ml of physiologic solution, 1000 ml of glucose solution, antibiotic prophylaxis (Kefazol 1 g 8/8 h), analgesics and antiemetics, if needed. Laboratory tests were redone, and the presence of nausea and flatus were monitored during this time. In the 48 hours postoperative period, patients from this group started a restricted liquid diet 36 hours after the surgery and were prescripted 1000 ml of physiological solution, 1000 ml of glucose solution, analgesics and antiemetics - if needed. Once again, laboratory tests were redone, and nausea and flatus were monitored during this time.

### Statistical analysis

This study analyzed variables like mean, median, standard deviation, minimum and maximum values. The resume of qualitative variables was performed considering frequencies and percentages. Aiming to compare the two groups concerning quantitative variables, Student’s t-test for independent samples was used. The Chi-Square test or Fisher’s exact test was used to compare the two groups concerning qualitative variables. Nausea and flatus comparisons between the groups were performed by binomial teste. Split-Plot analysis of the variance model was used to compare the time of evaluation. Bonferroni test was applied in the groups comparison within each moment and the moments comparison within each group. P values ​​less than 0.05 indicated statistical significance.

## RESULTS

Table 1 shows that the two groups were homogeneous regarding age, weight, height, BMI and surgical time.

**TABLE 1:** Group homogeneity

Variable	Group	n	Mean	Median	Minimum	Maximum	Standard deviation	p
Age	Ringer Lactae	40	39.8	39.5	20.0	68.0	10.0	0.121
	Physiologic Sol.	40	36.4	33.5	20.0	55.0	9.5	
Weight	Ringer Lactate	40	111.9	110.0	92.0	137.0	13.7	0.307
	Physiologic Sol.	40	114.9	117.0	87.0	139.0	12.4	
Height	Ringer Lactate	40	1.63	1.63	1.46	1.86	0.09	0.897
	Physiologic Sol.	40	1.63	1.63	1.50	1.76	0.06	
BMI	Ringer Lactate	40	41.86	41.48	35.82	45.76	2.52	0.020
	Physiologic Sol.	40	43.13	43.98	38.67	45.94	2.26	
Surgery time (min.)	Ringer Lactate	40	93.0	95.0	60.0	115.0	14.5	0.410
	Physiologic Sol.	40	90.0	90.0	60.0	145.0	17.2	

(*)Student’s t-test for independent samples; p<0.05

There were no episodes of bronchoaspiration, and there were no gastrojejunal fistulas in either group.

For the quantitative analysis of the residual gastric volume (RGV) in the PS and RL, the means were respectively 16.5 and 8.8 (p<0.001, Table 2).

**TABLE 2:** Groups comparison related to quantitative analysis of RGV

Variable	Group	n	Mean	Median	Minimum	Maáximum	Standard deviation	p
RVG	Ringer Lactate	40	8.8	8.5	0.0	19.0	4.6	<0.001
	Physiologic Sol	40	16.5	17.0	0.0	32.0	9.2	

(*) Student’s t-test for independent samples; p<0.05

### Clinical analysis

#### Na (sodium)

Sodium was higher in PS patients after the surgery compared to RL’s patients but without statistical significance. In the first 24 hours, a constant difference between them was noticed, however with statistical significance (p=0.013). After 48 hours, there was an increase in the RL group mean value - which reached 139.25 - but without significance compared to the PS, which had a mean of 138.98.

#### K (potassium)

By the end of the operation, the K serum level of the PS groups had a mean of 3.83, whilst for the RL group, the mean was 3.64 (p<0.029). Such a significant difference was not observed in subsequent analyses. RL showed an increase from its initial levels during the first 24h (p=0.016). In sequence, was observed a fall on these values below the initial point, again demonstrating statistical significance between values of 24 h and 48 h. PS group patients in the immediate postoperative period up to 48h had decreased levels with statistical significance (p<0.001).

#### Urea, creatinine and CPK (creatine phosphokinase)

In the immediate postoperative period, both groups had equivalent serum levels, with no significant difference. However, in the 24 h, both had an increase and significant difference in the 48 h with a decrease in values (p<0.001).

#### Insulin

The values found in the PS were higher and presented a significant difference if compared to the RL in the first 24 hours. Afterwards, the insulin levels were reduced close to the initial ones (p=0.536) if compared to themselves, but showing a significant difference for both groups compared to the insulin values between 24 and 48 h.

#### pH

In the immediate postoperative period, the pH was 7.36 for the PS and 7.39 for the RL without statistical significance. In the following moments, were not observed any statistical significance for this parameter.

#### Sodium bicarbonate (SB)

The bicarbonate level showed a significant difference between the groups at all moments, being more expressive in the immediate postoperative period; PS was at 22.37 while 24.26 showed in RL (p<0.001). An increase was seen in the 24h period evolution of PS, with a posterior decrease in the 48h period. It has shown statistical significance if compared to the RL group (p<0.016). 

#### PaCO2

There was a significant difference (p<0.040) between the groups in the first 24 h, where values were 40 (RL) and 37.55 (PS). Both groups had a decrease in values between the immediate and the 48 h period, with statistical significance (p<0.001).

#### Nausea

The incidence of nausea was 17.5% in the PS and 7.5% in the RL immediately after surgery and without statistical significance in the other periods.

#### Flatus

Both groups had flatus within the first 24 h. In RL, it represents 25% of patients, whilst 12.5% in PS. Although, this difference was not statistically significant. For the following 48h, it occurred in 62.5% of both groups, also without statistical significance.

## DISCUSSION

Evidence-based medicine is translated into practice in a context of integrated clinical experience, with the ability to critically analyze and rationally apply scientific information, aiming to improve the quality of medical care^15^. In this context, translational medicine is an area that studies how to accelerate innovations with the transmission of knowledge from basic research to clinical application, aiming the improvement of results in medical or surgical treatment^10^ ^,^ ^20^.

Literature has shown safety in fasting abbreviation offering maltodextrin for two hours before anesthetic induction^7^ ^,^ ^20^. A systematic review of 19 different preoperative fasting guidelines recommended avoiding prolonged fasting and attested safety in prescribing clear fluids up to two hours before the beginning of the anesthetic procedure. In order to change this paradigm about postoperative and postoperative fasting abbreviation, new guidelines have come up (ERAS and ACERTO Project), which comprise a series of evidence-based diligence performed by a multidisciplinary team during the pre, peri and postoperative periods. The application of these procedures aims to shorten the hospital stay, reduce complications and postoperative morbidity, recover and improve the patient’s quality of life. It is recommended that health professionals re-evaluate the behavior of long periods of fasting and change them to the abbreviation with the offer of a carbohydrate-enriched solution up to two hours before the operation. This practice has been shown to be one of the main factors to reduce insulin resistance and improve well-being. Thus, oral nutrition and/or early postoperative enteral nutrition seem safe and favors intestinal anastomoses healing^11^. 

Regarding this discussion in the literature, our work was based on a comparison between two groups. One with patients who underwent a long period of 12 hours fasting with fluid and solids restriction and a diet of 36 hours after the surgical procedure (PS group), and another did a 2 hours preoperative fasting, which consisted of a liquid carbohydrates diet and with a 24 hours diet after the surgical procedure (RL group). Patients who underwent long fasting periods (PS) had higher insulin levels in the first 24 hours (above reference values) compared to those who abbreviated their fasting for 2 hours before surgery (RL), which characterized an increase in insulin resistance. Aguilar Nascimento, et al. (2011)^3^ demonstrated that abbreviating the preoperative fasting up to 3 hours managing fluid carbohydrates and whey protein diet minimizes postoperative insulin resistance and reduces the magnitude of the acute-phase inflammatory response.

Another study reviewed the abbreviation of preoperative fasting from the metabolic and gastric emptying points of view, clinical benefits, and current recommendations^11^. According to Campos, fasting abbreviation with a carbohydrate-enriched drink up to 2 hours before the surgical procedure can benefit glycemic and functional parameters, reduce hospitalization, and have no risk of bronchoaspiration in healthy individuals undergoing elective operations. Another nutrient often added to this carbohydrate solution, with promising results, is glutamine. The abbreviation of preoperative fasting with carbohydrates and glutamine enriched drinks seems to be effective in the care of surgical patients and optimize the recovery of the postoperative period^11^. In this study, patients who had fasting abbreviation of 2 hours, with carbohydrate-enriched drink and early feeding (RL) had lower RGV values, lower insulin resistance, lower incidence of nausea in the immediate postoperative period, shorter hospital stay and better postoperative recovery, compared to the group that remained in prolonged fasting preoperatively and diet after 36 hours (PS). Both groups did not show signs of fistula and bronchoaspiration.

Yagci, et al. (2008)^30^ evaluated the effects of preoperative carbohydrate loading on patients’ gastric content and perioperative metabolism. Those in the treatment group (n=34) received 800 ml of a carbohydrate-rich liquid the night before the operation and 400 ml of the same liquid 2 hours before the procedure. On the other hand, patients in the control group (n=36) underwent overnight fasting. Plasma glucose and serum insulin levels were obtained during the perioperative period and anesthetic induction. The volume and pH of the preoperative residual gastric contents were also measured. Preoperative plasma glucose levels were found to remain significantly higher in patients who received the carbohydrate-rich fluid. Serum insulin levels initially elevated in the study group returned to control levels at the time of induction of anesthesia. There was no statistical difference between the two groups in terms of gastric content or gastric liquid pH. They concluded that preoperative ingestion of fluids rich in carbohydrates does not seem to change the volume or the pH of gastric contents, suggesting that this is a safe procedure in terms of risk of aspiration. In addition, drinking this liquid can prevent energy absence. In the present study, RL patients abstained from solid food for 6 hours and had a 50 g of maltodextrin in 100 ml of mineral water solution, which were administered 2 hours before the beginning of anesthesia. At the same time, the PS group abstained from solids and liquids for 12 hours. In RL, we obtained a lower serum insulin value in the first 24 hours. Unlike Yagci, et al. (2008)^30^, we obtained a lower mean of the RGV if compared to the PS patients. Furthermore, there was no episode of bronchoaspiration in either group.

According to Yagci et al. (2008)^30^ and Itou, et al. (2012)^17^, we performed a multicenter trial to investigate the safety and efficacy of oral rehydration therapy up to 2 hours before the operation, using an oral rehydration solution. They concluded that gastric volume immediately after anesthesia induction was equal in both groups, and no significant difference in pH was found. Thus, this study supports the safety of oral rehydration therapy up to 2 hours before surgery.

Ruiz-Tovar J, et al (2019)^24^ evaluated postoperative pain, nausea or vomiting. One hundred eighty patients were included in the study, 90 in each group. Postoperative pain (16 vs. 37 mm; p<0.001), nausea or vomiting (8.9% vs. 2.2%; p=0.0498) and hospital stay (1.7 vs. 2.8 days; p<0.001) were significantly lower in the ERAS group. This group was associated with less pain, lower incidence of nausea or vomiting, lower levels of acute-phase reactants and early hospital discharge, in agreement with the present study, which compared RL and PS groups in the immediate postoperative period. As a result, the incidence of nausea was 7.5 (RL) and 17.5% (PS).

Preoperative overnight fasting was introduced when anesthetic techniques used chloroform and were still very rudimentary to avoid respiratory complications resulting from vomiting and aspiration of gastric contents. In our study, patients in both prolonged and 2-hour fasting groups did not show signs of bronchoaspiration.

Insulin resistance is the primary metabolic change during surgical stress, directly proportional to the magnitude of the operation and causes hyperglycemia in non-diabetic patients. As a result, various endocrine and inflammatory systems are stimulated. This results in an intensified postoperative catabolic response, which implies losing body fat and protein reserves. In addition, aggressive insulin treatment to maintain normal glycemic levels resulted in reducing organ dysfunction and mortality. Moreover, insulin resistance showed to be an independent factor influencing hospitalization time^7^.

Six clinical trials demonstrated a significant reduction in insulin resistance after using preoperative carbohydrate loading. The maximum improvement in insulin action observed was a factor of 50% (p<0.01) after the morning carbohydrate dose on the day of operation^7^. In the present study, corroborating these authors, lower serum insulin levels were obtained in RL group, which demonstrates lower insulin resistance in the same group and differs from PS group - which had prolonged fasting.

Matłok, et al. (2016)^22^ assessed the risk of rhabdomyolysis after bariatric surgery related to the administration of intravenous fluids in the perioperative period. A total of 194 patients underwent laparoscopic sleeve gastrectomy. The mean operative time was 132 min. The mean volume of fluids administered was 3150 ml, from the anesthetic induction until 24 hours later. Biochemical rhabdomyolysis (creatine phosphokinase >1000 U/l) was observed in 30 patients (15.46%). Rhabdomyolysis with clinical manifestations developed in six patients. Therefore, there was an association between the administration of smaller fluid volumes and a lower risk of rhabdomyolysis. It is postulated that decreasing intravenous fluid administration may reduce the risk of rhabdomyolysis after bariatric surgery. Our study corroborates Matlok, et al. (2016)^22^, in which all patients underwent up to 120 min of surgery time. Those who had fasting abbreviation to 2 hours with carbohydrates and fluid restriction in the transoperative period had lower levels of CPK in the first 24 hours, compared to those who had prolonged fasting and greater fluid intake in the transoperative period. However, none of these groups had high levels of CPK to characterize rhabdomyolysis.

The surgical and anesthesiological practice is under constant development, which means that the recommendations in the literature need to be questioned and updated^25^ ^,^ ^29^. There are reasons to believe that successful implementation of ERAS programs will lead to reduced hospital costs. A recent review concluded that ERAS protocols appear clinically and economically efficient, but studies reporting out-of-hospital cost data are lacking. For this reason, more research is needed to determine the best way to assess the reduction in these costs provided by the ERAS protocol, taking into account quality of life data^16^.

## CONCLUSIONS

The abbreviation of preoperative fasting and early postoperative refeeding on Roux-en-Y gastrojejunal bypass with the application of programs such as ERAS or ACERTO Project accelerated the patient’s recovery, reducing residual gastric volume and insulin level, and it does not predispose to complications.
